# Applying TADF Emitters in Bioimaging and Sensing—A Novel Approach Using Liposomes for Encapsulation and Cellular Uptake

**DOI:** 10.3389/fchem.2021.743928

**Published:** 2021-09-01

**Authors:** Poppy O. Smith, Dominic J. Black, Robert Pal, João Avó, Fernando B. Dias, Victoria L. Linthwaite, Martin J. Cann, Lars-Olof Pålsson

**Affiliations:** ^1^ Department of Chemistry, Durham University, Durham, United Kingdom; ^2^ IBB-Institute for Bioengineering and Biosciences, Instituto Superior Técnico, Universidade de Lisboa, Lisboa, Portugal; ^3^ Department of Physics, Durham University, Durham, United Kingdom; ^4^ Department of Biosciences, Durham University, Durham, United Kingdom

**Keywords:** fluorescence microscopy, bioimaging, liposomes, thermally activated delayed fluorescence (TADF), sensing

## Abstract

A new method for facilitating the delivery, uptake and intracellular localisation of thermally activated delayed fluorescence (TADF) complexes was developed. First, confinement of TADF complexes in liposomes was demonstrated, which were subsequently used as the delivery vehicle for cellular uptake. Confocal fluorescence microscopy showed TADF complexes subsequently localise in the cytoplasm of HepG2 cells. The procedures developed in this work included the removal of molecular oxygen in the liposome preparation without disrupting the liposome structures. Time-resolved fluorescence microscopy (point scanning) showed initial prompt fluorescence followed by a weak, but detectable, delayed fluorescence component for liposomal TADF internalised in HepG2 cells. By demonstrating that it is possible to deliver un-functionalised and/or unshielded TADF complexes, a sensing function for TADFs, such as molecular oxygen, can be envisaged.

## Introduction

**FIGURE 1:**
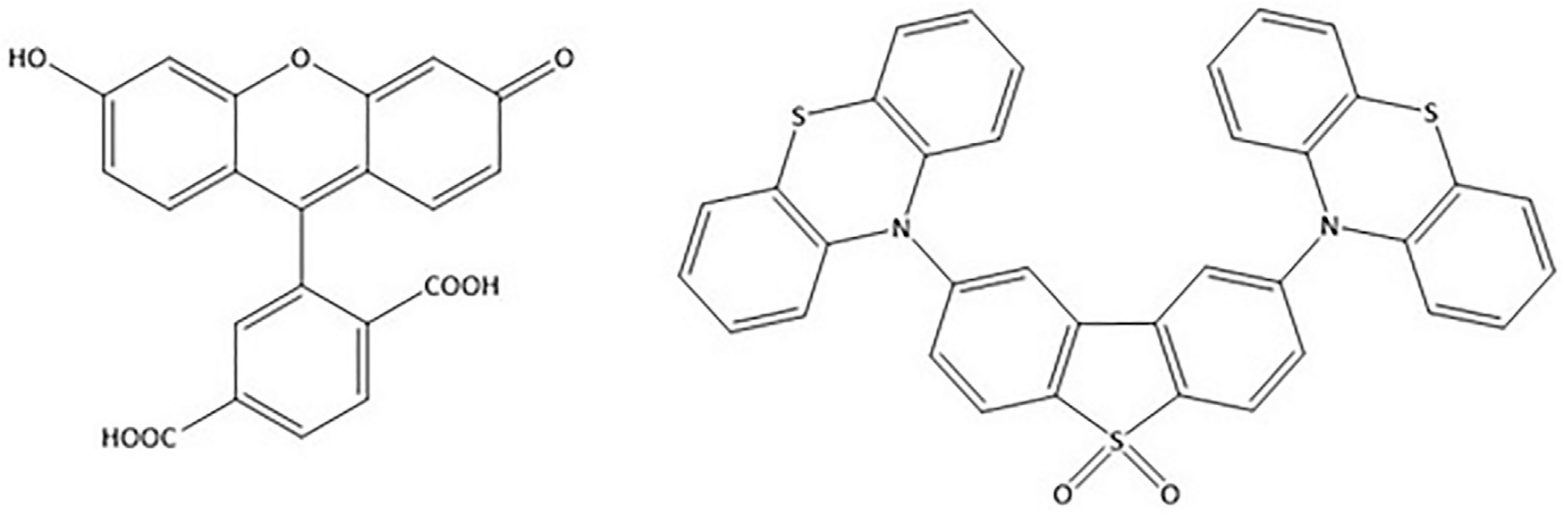
Chemical structure of molecular systems and complexes used in this work. 5 (6)-Carboxyfluorescein **(left)** and the TADF complex (2,8-di (10H-phenothiazin-10-yl)dibenzo [b,d]thiophene 5,5-dioxide) (DPTZ-DBTO2) **(right)** in their skeletal formula. See text for details.

Fluorescence-based bioimaging techniques are widely used to study the structure and function of bioactive systems in real-time. Nowadays, these techniques can offer high spatial resolution in combination with non-invasive approaches, key in live-cell imaging (Nagano 2010; Morris, 2014; Stockert and Blazquez-Castro 2017). The application of fluorescence-based bioimaging requires the use of fluorescent optical probes or sensors. The most commonly used fluorescent probes include purely organic materials with fluorescent lifetimes in the ns regime, such as low molecular weight probes and recombinant proteins (Patsenker et al., 2008; Volkmer et al., 2000). However, in confocal fluorescence microscopy, the use of such fluorescent probes can be problematic due to the presence of endogenous chromophores. These will produce auto/background fluorescence, in particular under UV-blue excitation, resulting in poor image contrast. The most successful approach to overcome this problem is time-gated detection of photoluminescence (PL). In this endeavor, optical emissive probes with emission lifetimes in the μs—ms regime are required. The classes of emissive probes used in this context are based on metal centred emission (functionalised lanth complexes) and/or organometallic (transition metal) complexes (Rajendran and Miller 2015; Zhao et al., 2011; Ma et al., 2013). While there have been some significant achievements in bioimaging using these classes of materials, pertinent questions regarding the use of scarce and expensive rare earth and transition metals remain unanswered. A new class of purely organic materials, with the key feature of long-lived emission in the μs—ms regime, is now emerging as powerful alternatives to lanthanide and organometallic complexes for use in bioimaging. These are the so-called thermally activated delayed fluorescence (TADF) emitters (Dias et al., 2017). TADF emitters have found extensive use in display applications, as organic light-emitting diode (OLED) materials, due to the exploitation of their high triplet state harvesting, to design bright OLEDs with excellent device characteristics (Uoyama et al., 2012, Zhang et al., 2012, Penfold et al., 2018, Li et al., 2014). The principal operation of a TADF emitter is based on a relatively small singlet-triplet energy gap (Δ*E*_ST_), close to *k*_*B*_ *T* (*k*_B_ *T* ∼ 200 cm^−1^ at ambient temperatures). Therefore, within the excited state lifetime, there can be an uphill electronic transition of reverse intersystem crossing (RISC) from the lower-lying triplet state back to the singlet state, from which the emission (in principle fluorescence) can occur. These excited state electronic transitions can lead to E-type delayed fluorescence (DF) with dynamics in the μs—ms range in parallel to the prompt fluorescence (PF) with its typical ps—ns dynamics (Dias et al., 2017). The DF is, therefore, the desirable emission component for time-gated bioimaging in this context. Furthermore, as the triplet state is sensitive to molecular oxygen, which in principle can quench the DF, the interplay or relative intensities between PF and DF provides a sensing functionality yet to be fully exploited (Méhes et al., 2012). Although the optical properties of TADF emitters are desirable for bioimaging applications, the biocompatibility of TADF emitters with the intracellular environment is generally poor. Additionally, most of the TADF complexes designed to date are not well suited for aqueous environments, although there are some exceptions (Yin et al., 2020). Moreover, the polar intracellular environment would significantly reduce both PF and DF, with molecular oxygen having an additional detrimental impact on emission intensity. Accordingly, there is a need for further functionalization of TADF emitters to improve their biocompatibility. Thus far, the approaches taken have included various encapsulation methods, intending to shield the TADF emitter from the cellular environment whilst enhancing cellular uptake and retention (Xiong et al., 2014, Li et al., 2017, Qi et al., 2020 Luo et al., 2020, Crucho et al., 2020). In this work, we developed a novel method for TADF emitter confinement and cellular delivery/uptake based on liposomes. We elected to use (2,8-di (10H-phenothiazin-10-yl)dibenzo [b,d]thiophene 5,5-dioxide) (DPTZ-DBTO2) (Figure 1) to test our hypothesis of using liposomal structures as a delivery tool for bioimaging. DPTZ-DBO2 was developed primarily as an OLED material (Dias et al., 2016) and if this system can be used in bioimaging, it is reasonable to assume that other TADF emitters could be employed in bioimaging applications. The viability of using liposomes for this purpose was demonstrated using a standard organic fluorescent dye molecule as a reference system. Furthermore, cellular uptake and retention of liposomal TADF was achieved, and time-resolved fluorescence microscopy showed DF from liposomal TADF in cellulo.

## Results and Discussion

**FIGURE 2:**
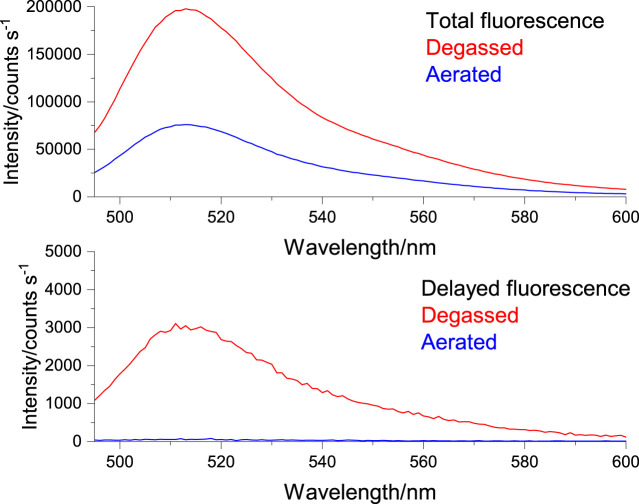
Degassed (red) and aerated (blue), total fluorescence (upper panel) and delayed fluorescence (lower panel) from liposomal DPTZ-DBTO2 emitters from PD-10 separated fraction 1 (Supplementary Figure S2), 5 mM DPTZ-DBTO2 emitter 100 nm liposomes. Excitation is 390 nm, emission 495–595 nm. Delayed emission (lower panel) collected 10 μs after excitation. See text for detail.

**FIGURE 3:**
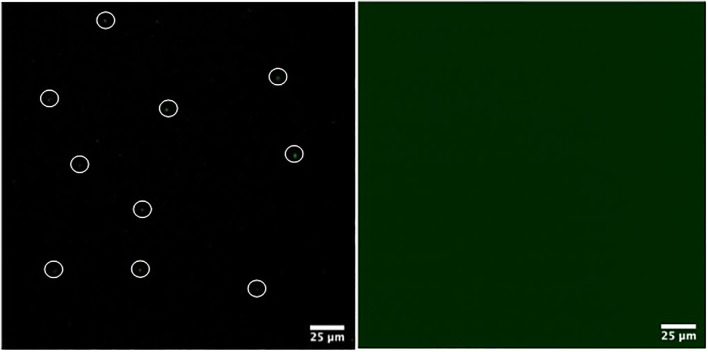
Comparison of liposome packaged (left panel) and free DPTZ-DBTO2 (right panel). Left panel showing liposome packaged DPTZ-DBTO2 (some circled for clarity), from fraction 1 (Supplementary Figure S2), imaged 30 min after PD-10 desalting column separation, once freeze-pump-thawing had been performed. Packaged and free DPTZ-DBTO2 complex gave the same fluorescence intensity count. See text for details.

**FIGURE 4:**
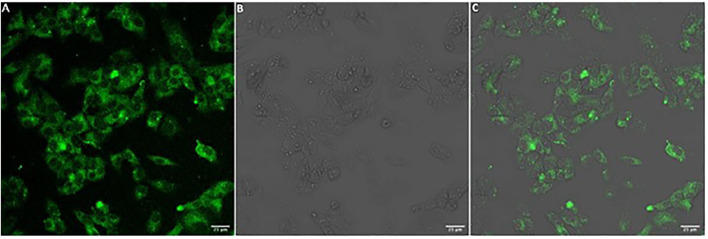
HepG2 cellular introduction of liposomal DPTZ-DBTO2 under 24 h incubation with 20% v/v PD-10 column separated liposomes from fraction 1, with liposomes 100 nm in diameter. Confocal microscopy images for **(A)** DPTZ-DBTO2 emission, **(B)** bright field and **(C)** DPTZ-DBTO2 emission and bright field overlaid. Scale bar (bottom left, white rectangle) 25 μm in length. See text for details.

**FIGURE 5:**
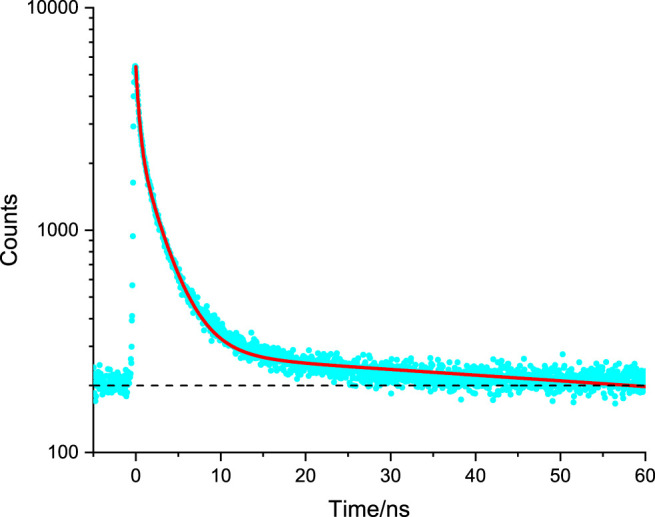
Representative time-resolved fluorescence microscopy decay of NIH 3T3 internalised Amine-Functionalized DPTZ-DBO2 doped Nanoparticles, used to determine fluorescence lifetime by fitting the data (blue dots) to a curve (red line) through least-squares fitting procedures. Dashed black line indicates a baseline intensity. See text for details and Table 1 for data.

Due to the inherent sensitivity of molecular oxygen on the TADF emission, particularly the DF component, oxygen removal procedures must be applied on liposomal TADF emitters. However, in conjunction, it is necessary to check the integrity and intactness of the liposomal structures after the oxygen removal procedures. This reason was the rationale behind using (6)-Carboxyfluorescein (CF) (Figure 1) as our reference system (Supplementary Figure S3). We elected to use a freeze-thaw strategy (using liquid N_2_) with five cycles to ensure efficient molecular oxygen removal. For this purpose, liposomes were collected in the separation procedure (Supplementary Figure S1) and investigated using a combination of fluorescence spectroscopy on liposome solutions, and confocal fluorescence microscopy on slide deposited liposomes (Supplementary Figure S4). We could then proceed and apply this protocol to obtain liposomal TADF particles. (Supplementary Figure S2) shows the fluorescence of liposomal DPTZ-DBTO2 in solution. The decrease in intensity of the total fluorescence for the aerated sample (relative to the degassed sample) was expected, as the DF component would be affected due to molecular interactions between DPTZ-DBTO2 and molecular oxygen, leading to triplet state quenching. The lower panel in Figure 2 shows the DF component is still present in the degassed solution, proven by the fact that aerating the previously degassed solution results in the complete extinction of the DF. Confocal fluorescence microscopy showed the liposomal structures could proceed through the freeze-thaw procedures and remain intact. Liposomal CF was used to test this hypothesis, and confocal images show emissive particles with a size of ∼100 nm in the CF spectral range (Supplementary Figure S3). The key observation made was that emissive particles with similar size and brightness were observed after the freeze-thaw procedure had been applied (Supplementary Figure S3). Figure 3 shows confocal images of emissive particles attributed to liposomal DPTZ-DBTO2 from a solution that underwent the freeze-thaw procedure. As a control, the right panel of Figure 3 shows the confocal image obtained from a solution of similar DPTZ-DBTO2 content but collected from the first fraction of the separation column. No emissive particles can be observed; the emission is of uniform intensity across the image and is therefore attributed to free DPTZ-DBTO2. Cellular uptake, retention and localisation of liposomes containing fluorescent probes were tested on HepG2 cells. The uptake was at first tested using liposomal CF (Supplementary Figure S6). From this, optimal conditions were determined by assessing the confocal images obtained for liposomal CF, identifying the best conditions for the uptake of liposomal TADF systems. To check the localisation profile, Hoechst 33342 was used as a co-stain; this probe typically locates in the cellular nuclei, staining DNA (Mocharla et al., 1987). Particle size appeared to be important for uptake by HepG2 cells (but also generally) because endocytosis of particles larger than 200 nm diameter was problematic. We, therefore, elected to use particles of the size 100 nm diameter for probe intracellular internalization. Supplementary Figures S7,8 (supplementary information) shows the results of the trial with different particle sizes. For liposomal CF, the fluorescence did not occur or originate from the nucleus. The CF was, therefore, most likely localized to the cytoplasm. Furthermore, the CF fluorescence was even across the cytoplasm, suggesting the liposome fused with the cell membrane, subsequently releasing the probes into the cytoplasm. Cellular introduction of liposomal DPTZ-DBTO2 was achieved with 100 nm diameter liposomes at 20% v/v PD-10 column separated liposomal probe concentration, under 24 h incubation (Figure 4 and Supplementary Figure S9). Fluorescence intensity and distribution of intracellular DPTZ-DBTO2 appeared greater and more uniform than intracellular CF (Supplementary Figure S8). This may have been due to more DPTZ-DBTO2 containing liposomes internalised per cell, permitted by the increased cell membrane surface area exposed to the extracellular environment because of the less tightly packed cell clusters and lower cell confluency. Images from cell cluster packing (Supplementary Figure S8) of closer similarity gave fluorescence intensity and distribution more comparable to CF (Supplementary Figure S8). This indicated cell confluency could be used to control the extent of probe uptake, in addition to the concentration of liposomal probe solution. Like CF, internalised DPTZ-DBTO2 emitters were uniformly distributed throughout the cytoplasm with fluorescence outlining the nuclei, unable to cross the nuclear envelope. This suggested liposome fusion to the cell membrane had occurred, releasing probe contents intracellularly, as opposed to entire liposome cellular internalization. However, the intracellular probe DPTZ-DBTO2 appeared non-aggregated despite being present in the aqueous cellular environment. However, no Hoechst 33342 nuclei staining was used in the cellular introduction of DPTZ-DBTO2 shown in Figure 4 because of an ambiguity caused by Hoechst 33342 emission in the detection of internalised DPTZ-DBO2 doped nanoparticles in NIH 3T3 cells (Supplementary Figure S5). By omitting Hoechst 33342 staining, most of the fluorescence detected upon visible excitation was attributed to DPTZ-DBTO2, with a minor autofluorescence contribution. The intracellular localisation profile of TADF emitters is not clear; there are only a limited number of studies using TADF emitters in bioimaging to date, and in some of those previous works, encapsulation of the TADF emitter to form nanostructures have been pursued as the key objective (Li et al., 2017, Qi et al., 2020 Luo et al., 2020). The rationale for that approach has primarily been driven by the desire to minimise interactions between TADF emitters and molecular oxygen. Furthermore, a study describe how BSA can be used as an intracellular delivery system, with the same rationale of molecular oxygen shielding (Xiong et al., 2014). To our knowledge, only in the work by Yin *et al.* was uptake of an un-functionalized TADF emitter demonstrated (Yin et al., 2020). This TADF system is unique as it can function as an intracellular sulfite sensor (Yin et al., 2020). The relatively small body of previous works shows intracellular localisation in lysosomes (Xiong et al., 2014; Qi et al., 2020), in most cases, but also in the cytosol (Crucho et al., 2020) and on the cell membrane (Li et al., 2017). The unique and unusual time domains of the TADF emission remain the centerpiece, driving the development of TADF systems for bioimaging. In this present work, point scanning of the same sample materials (as used in the confocal imaging) was performed using a time-correlated single photon counting (TCSPC) system built onto an epifluorescence microscope (Pal et al., 2018). This was motivated by the superior signal-to-noise ratio and excellent sensitivity of that detection system. Admittedly, it is not practically feasible to fully monitor the emission in the μs time domain with our TCSPC systems. However, the time window was stretched to 110 ns in an attempt to reveal contributions from any long-lived decays phases. In this part of the work, we also investigated polystyrene nanoparticles containing the TADF emitter DPTZ-DBTO2, internalised in NIH 3T3 cells. The application of these nanoparticles in bioimaging was also described by Crucho et al., 2020, but for MCF-7 cells. Confocal images of DPTZ-DBTO2 nanoparticles in NIH 3T3 cells can be found in the SI (Supplementary Figure S5). As alluded to earlier, there is some ambiguity concerning these images due to the interference by nuclei stain Hoechst 33342, and the localisation profiles are therefore somewhat uncertain. However, the time-resolved emission gave a clearer picture concerning both the nuclei stain and the DPTZ-DBO2 nanoparticles. Moreover, the time-resolved fluorescence microscopy provides an opportunity to compare the fluorescence decay between functionalised encapsulated TADF nanoparticles and liposomal TADF emitters. The emission decay (Figure 5) is best fitted to a sum of three exponential terms (Table 1 for data). The first sub-nanosecond component is difficult to assign uniquely to any of the optical probes internalised in the cell. The most likely explanation is that this decay phase is autofluorescence from endogenous chromophores. For instance, NADPH produces fluorescence with a lifetime in the 500 ps range in the same spectral region (Ogikubo et al., 2011). However, we remark that there could also be a contribution of PF from the DPTZ-DBO2 nanoparticles. The yield of each component was calculated according to,

$$y_{i}=\frac{a_{i}\tau_{i}}{\sum_{i}a_{i}\tau_{i}}$$ (1)

**TABLE 1:** Data obtained from time-resolved fluorescence microscopy and fitted to a sum of exponentials (Eq. 1).

Delivery system	Exp. Term	*τ*_i_/ns	*Α*_i_/a.u	*Yield*_i_/%
Nano particles	1	0.42 ± 0.01	3,130.9 ± 31.3	14.4 ± 0.1
	2	2.35 ± 0.05	2162.2 ± 24.5	55.0 ± 4.4
		3	10.20 ± 0.60	277.4 ± 23.6	30.6 ± 4.4
	Liposomes	1	0.66 ± 0.06	874.5 ± 148.3	4.2 ± 0.9
2		4.20 ± 0.28	831.7 ± 105.2	25.6 ± 3.6
		3	62.32 ± 1.73	153.4 ± 15.1	70.1 ± 4.5

Data presented here is based on several acquisitions at the different positions on the slides and from different slides. Yields for individual components calculated according to Eq. 2 where *τ*_i_ = (*k*_i_)^−1^ (Eq. 1). See text for details.

**FIGURE 6:**
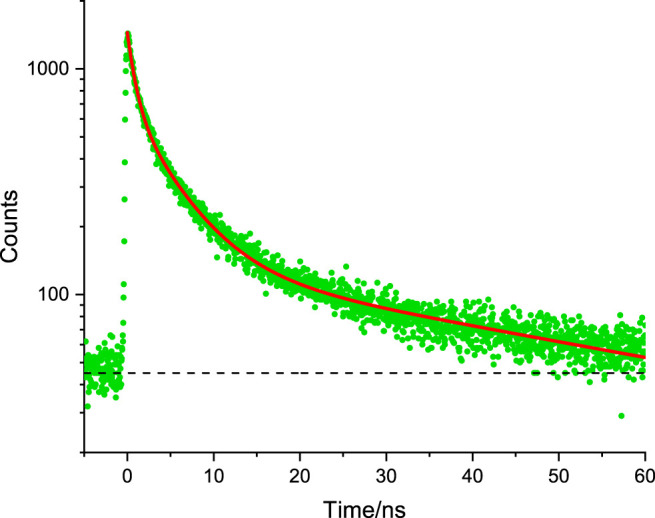
Representative time-resolved fluorescence microscopy decay of HepG2 internalised liposomal DPTZ-DBO2 used to determine fluorescence lifetime by fitting the data (green dots) to a curve (red line through least-squares fitting procedures. Dashed black line indicates a baseline intensity. See text for details and Table 1 for data.

It is noteworthy that the relative contribution of the sub-nanosecond phase component is low as the calculated yield is the smallest in the overall decay for internalised DPTZ-DBO2 nanoparticles (Table 1). The second decay phase in the emission from labelled NIH 3T3 cells is attributed to the nuclei stain Hoechst 33342. This is in line with what has been reported previously (Crucho et al., 2020; Okkelman et al., 2020). The third, and most long-lived decay phase, is attributed to PF from DPTZ-DBO2 nanoparticles, in line with the observation by Crucho et al., 2020, who reported a very similar PF lifetime for the same type of DPTZ-DBO2 nanoparticles (Crucho et al., 2020). Consequently, we cannot claim there is a DF component in the emission from DPTZ-DBO2 nanoparticles internalised in NIH 3T3 cells, based on the fit of the data in Figure 5. The spectroscopy experiments carried out by Crucho et al., 2020 resulted in PF lifetimes in the ns range, 3.0–22.0 ns, and DF lifetimes in the range of 0.97–39.1 µs, depending on the functionalization of the nanoparticles (Crucho et al., 2020). The longer DF lifetimes observed by Crucho et al., 2020 is outside the measurement range of our TCSPC detection system. Furthermore, based on observations by Crucho et al., 2020, from the same DPTZ-DBO2 nanoparticles, the intensity of the DF component would very likely be 2–3 orders of magnitude weaker than the PF component (Crucho et al., 2020). This would make the detection of a DF component very challenging in a fluorescence microscopy experiment. However, in an expanded time window there appears to be a very weak emission tail from the preceding excitation event, still decaying 0.9 μs after excitation, possibly due to a very weak DF component, as seen in Supplementary Figure S10. By contrast, it was observed by Li et al., 2017 that the DF component of TADF quantum dots is extinct *in cellulo*, which was explained by molecular interaction with molecular oxygen. This is a likely explanation for the DPTZ-DBO2 nanoparticles as the polystyrene functionalization cannot fully shield the chromophores against water and/or oxygen (Horak et al., 1998). For liposomal DPTZ-DBO2 emitters, internalised in HepG2 cells (as previously described), the time-resolved emission leads to slightly different results (compared with the DPTZ-DBO2 nanoparticles in NIH 3T3), see Figure 6. Also here, three exponential terms were required to obtain the optimal fit of the data (Table 1). For HepG2 cells, only liposomal DPTZ-DBO2 was applied, as previously stated. However, this only leads to a minor change in the interpretation of the first two faster decay phases. The fast component is, as before, attributed to autofluorescence, presumably from NADPH. Similarly, the second decay phase is attributed to PF from DPTZ-DBO2. The third most long-lived component (63.2 ns) is, interestingly here, also the dominating decay phase according to the yield calculated, primarily due to its long fluorescence lifetime. In this case, we, therefore, propose there are DF contributions to this decay phase, although we admit that our experimental apparatus cannot fully detect emission lifetimes in the μs range. In the work by Crucho et al., 2020 there is, however, no PF component observed with such a long lifetime. The hypothesis of DF contributions to this decay phase is further supported by again considering the extended time window (Supplementary Figure S10). At long decay times, approximately 0.9 μs after the excitation event, the tail end of a μs decay phase can be seen, with a more significant intensity than in the case for DPTZ-DBO2 nanoparticles in NIH 3T3 cells. It is therefore possible that the long lifetime observed for liposomal DPTZ-DBO2 is underestimated, but not necessarily with a μs emission lifetime as some interaction with molecular oxygen could have occurred. We remark that we do not anticipate any significant differences in impact on the photo physics from the different intracellular environments of NIH 3T3 cells and HepG2 cells. Crucially though, the observation here suggests liposomal DPTZ-DBO2 emitters can enter cells and internalise without encountering an amount of molecular oxygen that would subsequently lead to a complete quenching of the DF emission.

## Conclusion

In this work, we demonstrated liposomes can be used as a vehicle for the delivery and internalization of TADF emitters and complexes in bioactive cells. The liposomal structures remain intact after the molecular oxygen removal procedure, involving repeated freeze-thaw steps. Thus, TADF emitters can be delivered to cells without encountering molecular oxygen and localize in the cytoplasm. In addition, time-resolved fluorescence microscopy provides strong indication for a DF emission component from liposomal TADF. The interplay between PF and DF in TADF emitters provides a sensing functionality, unique for this class of materials. Further work could therefore be devised to explore this sensing functionality of internalised TADF emitters, such as molecular oxygen in live-cell imaging.

## Experimental

### Materials

Fluorescent probes packaging was conducted to facilitate cellular probe uptake in a controlled manner. Fluorescent probe packaging protocols were initially developed with the model fluorescent probe: 5 (6)-carboxyfluorescein (CF) with MR 379.32 g mol-1 (obtained from Sigma Aldrich and used without further modification). The test probe CF chosen was readily available and atmospherically insensitive with a comparatively high brightness (Fahmy et al., 2017). Once established, probe packaging protocols were modified for use with the target TADF emitter; (2,8-di (10H-phenothiazin-10-yl)dibenzo [b,d]thiophene 5,5-dioxide) (DPTZ-DBTO2) with MR 610.76 g mol-1 (designed as described in (Gan et al., 2015; Dias et al., 2016)). The use of the model probe in the initial stages overcame the handling difficulties of DPTZ-DBTO2 caused by its lipophilic and oxygen-sensitive nature (Dias et al., 2017; Gan et al., 2015; Dias et al., 2016). Liposome formation was adapted from Vlasova et al., 2019 to incorporate probe addition at a point compatible with probe solubility. The general procedure, reverse phase evaporation followed by freeze-thaw, was kept consistent between different probes: a lipid film of soy PC (1.5 mg) and cholesterol (0.1 mg) were formed by combining both components in chloroform before removing the solvent in vacuo. The lipid film was hydrated by addition of phosphate saline buffer (PBS) solution (0.5 ml, 137 mM NaCl, 10 mM phosphate, 2.7 mM KCl, pH 7.4) and agitated using a vortex mixer to form a lipid dispersion. The dispersion underwent five freeze-thaw cycles (between -196 and 30°C) before extruding 10x through a polycarbonate filter (100 nm track-etched pores, Whatman Nucleopore) using a LIPEX thermobarrel extruder (Northern Lipids Inc., BC, Canada) at 55°C under a positive pressure of N_2_. A larger polycarbonate filter (400 nm track-etched pores, Whatman Nucleopore) was used to attain larger liposomes. CF is water-soluble at pH ≥ 5 (Fahmy et al., 2017) hence was incorporated into the liposome formation protocol by hydrating the lipid film with aqueous CF (0.5 ml, 5 mM in 10 mM PBS, pH 7.4). DPTZ-DBTO2 is lipid-soluble (Xiong et al., 2014) hence was incorporated into the liposome formation protocol by the addition of DPTZ-DBTO2 (0.5 ml, 5 mM in chloroform) to soy PC (1.5 mg) and cholesterol (0.1 mg) before solvent removal in vacuo. Furthermore, as DPTZ-DBTO2 was oxygen-sensitive, liposome formation was conducted under an inert argon atmosphere until lipid-probe dispersion extrusion. Liposomal CF was applied to a Sephadex G-25 PD-10 Desalting Column (GE Healthcare) pre-equilibrated with PBS (10 mM, pH 7.4) as detailed in the PD-10 Desalting Column Product Booklet (Code: 52130800), collecting 10 step-eluted 1 ml fractions, after the application of the full sample volume, using PBS (10 mM, pH 7.4) as the eluent (eluting the larger particles first). An additional two 1 ml fractions preceding the 10 fractions collected, as instructed by the PD-10 Desalting Column Product Booklet, were collected when developing the PD-10 Desalting Column separation protocol with CF. Separation of free and liposomal DPTZ-DBTO2 was performed as with CF. However, the fraction with the highest early intensity count for maximum emission wavelength was degassed by freeze-pump-thaw before use.

### Optical Spectroscopy

A Jobin Yvon Horiba Fluorolog-3^®^, fluorescence spectrometer was used for CW and time-gated fluorescence measurements. For CF, excitation was set to 480 nm and the scan range to 500–600 nm (step 1 nm, excitation slits 2.5 nm, emission sits 1.5 nm bandpass). For the TADF emitter DPTZ-DBTO2, excitation was set to 390 nm and the scan range to 495–595 nm (step 1 nm, excitation slits 2.5 nm, emission sits 1.5 nm bandpass). Time gated emission of TADF emitter DPTZ-DBTO2 was recorded 10 μs after excitation with 41 ms between flashes and 100 flash count. When the intensity count rose above 200,000 counts, a filter was installed between the sample and detector. Integration time was 0.1 s for five averaged spectra.

### Confocal and Time-Resolved Fluorescence Microscopy

Laser Scanning Confocal Microscopy images were taken on a modified Leica SP5 II microscope. CF were excited with the internal 488 nm laser line, and for and TADF emitters and the nuclei stain Hoechst 33342, the 355 nm line (attached UV laser) was used for excitation. Pinhole size was determined by Airy disc size calculated from the objective lens (HCX PL APO 63x/1.40 NA αBlue).

The liposomal probe sample (80 ml) images were taken from the PD-10 column fraction, which gave the highest early intensity count for the maximum emission wavelength. For comparison, an image of a liposome-free probe solution (80 μL), giving the same intensity count as the selected fraction, was also taken. Confocal microscopy was conducted on cells adhered to coverslips (13 mm diameter) grown in 24-well plates. Before adding a coverslip to sterile 24-well culture plates in Class II Biosafety Cabinet under sterile conditions, coverslips were sterilized with HCl (1 M, 24 h incubation), followed by a deionized H_2_O wash and an ethanol (70%) wash. Once air-dried under sterile conditions, a single coverslip was placed in each well of the 24-well plate. Cells (HepG2 cell line from American Type Culture Collection (ATCC) and NIH 3T3) were plated into the coverslip-containing sterile 24-well plates. 24 h after plating cells at 20% confluency, incubation with the liposomal probe from the PD-10 column fraction of the highest early intensity count for maximum emission wavelength was begun by gentle replacement of cell media (removed volume corresponding to the volume of liposomal probe solution added). Upon completion of the investigated liposome incubation time (48 h after cell plating), Hoechst 33342 nuclei stain (10X, 50 μL) was applied by incubation (10 min, 37°C) where required. The wells were gently washed with warmed (37°C) PBS (0.5 ml). Cells were fixed by incubation (10 min, 37°C) with formaldehyde (4% v/v, 300 μL). After formaldehyde removal, wells were gently washed with cold (4°C) PBS (0.5 ml). Coverslips were mounted onto a sterile glass slide on top of a varnished ring (10 mm diameter) using PBS (8 μL) as a mountant. Slides were stored (dark, 4°C) until use. CF was used to investigate the optimal liposome incubation conditions for the cellular introduction of liposome packaged fluorescent probes. A variety of incubation times (4, 8, 16 and 24 h), liposomal probe solution concentrations (20% v/v and 40% v/v) and liposome diameters (100 and 400 nm) were trialled in combination. Hoechst 33342 nuclei staining was used as a co-stain. The results of each condition were assessed by confocal microscopy. TADF emitter DPTZ-DBTO2 cellular introduction was conducted as with CF, with the discovered optimal incubation time (24 h), liposomal probe concentration (20% v/v) and liposome diameter (100 nm). No Hoechst 33342 nuclei stain was applied in parallel to DPTZ-DBTO2 (unless otherwise stated). Time-resolved fluorescence microscopy was performed on the same slides as prepared for confocal fluorescence (as described above). A home-built system was used for point scanning time-resolved fluorescence microscopy based on a Zeiss Axiovert 135M Inverted Epi-fluorescence microscope (Pal et al., 2018). The excitation source was the PicoQuant diode laser LDH-P-C-395, 70 ps pulses FWHM @ 1 MHz. A Zeiss 100x/1.4 NA oil immersion lens was used to detect the fluorescence selected using a 495 nm long-pass filter (Comar Instruments). The detection system is based on time-correlated single photon counting (TCSPC) with a photon counting module Idquantic id100–20 (www.idquantique.com/quantum-sensing/products/id100/) in combination with a Becker-Hickl SPC-130 TCSPC module. The data was subsequently fitted to a sum of exponentials;

$$F\left(t\right)=\sum_{i}A_{i}\exp\left(-k_{i}t\right)$$ (2)

by deconvolution with the instrument response function (IRF). The IRF in the time-resolved fluorescence microscopy was obtained through light scattering from Ludox particles dispersed on a microscope slide. The FWHM was ∼200 ps, which afforded a temporal time-resolution of ≥100 ps. The time window in the TCSPC was set to 110 ns with a pulse repetition rate of 1 MHz. This time window was the maximum time window practically possible, and with these parameters, the TCSPC can provide data with acceptable statistical robustness.

## Data Availability

The raw data supporting the conclusions of this article will be made available by the authors, without undue reservation.
