# Supplementary material for: Applying TADF Emitters in Bioimaging and Sensing—A Novel Approach Using Liposomes for Encapsulation and Cellular Uptake
Source: Front Chem. 2021 Sep 1;9:743928. doi: 10.3389/fchem.2021.743928 (PMC8440804; doi:10.3389/fchem.2021.743928)
Supplement: Supplementary file 1 [file DataSheet1.DOCX]

Supplementary Material

Applying TADF emitters in bioimaging and sensing – a novel approach using liposomes for encapsulation and cellular uptake

Poppy O. Smith^1^, Dominic J. Black^1^, Robert Pal^1^, João Avó^2^, Fernando B Dias^3^, Victoria L. Linthwaite^4^, Martin J. Cann^4^ and Lars-Olof Pålsson^1*^

^1^ Department of Chemistry, Durham University, Lower Mountjoy, Stockton Road, Durham, DH1 3LE, United Kingdom.

^2^ IBB-Institute for Bioengineering and Biosciences, Instituto Superior Técnico, Universidade de Lisboa, Portugal.

^3^ Department of Physics, Durham University, South Road, Durham, DH1 3LE, United Kingdom.

^4^ Department of Biosciences, Durham University, Stockton Road, Durham DH1 3LE, United Kingdom.

*** Correspondence:**Corresponding Author
[lars-olof.palsson@durham.ac.uk](mailto:lars-olof.palsson@durham.ac.uk)

# Supplementary Data Cell viability.

Cell viability analysis using trypan blue was performed manually as detailed in Stobbart et al. [M. J. Stobbart, Mammalian Cell Viability: Methods & Protocols, **2011**].

The trypan blue exclusion test, performed to determine cell viability percentage, concluded internalisation of liposomal TADF fluorophore was biocompatible and caused no membrane damage: liposomal TADF fluorophore incubated cells gave 97.3 ± 0.4% cell viability, insignificantly different to 97.1 ± 0.2% cell viability for cells incubated with the PBS control (uncertainty calculated by standard error). Both samples achievement viabilities were greater than 95%, indicative of a healthy cell stock. [F. Li, J. Qi, C. Qin, Z. Fu and W. Ren, *Oncol. Rep.*, **2018**, 40, 3743-3751].

# Supplementary Figures and Tables

## Supplementary Figures

**Supplementary Figure 1.** The relationship of 5(6)-carboxyfluorescein (CF) fluorescence emission intensity count with PD-10 fraction number measured using spectrofluorometer, with excitation 480 nm, emission 500-600 nm, taking intensity count at 517 nm for CF. From liposome formation protocol with 5 mM CF. Each marker represents an individual emission spectrum from which the intensity count at 517 nm (black) was obtained. A line links the markers.

**Supplementary Figure 2.** The relationship between peak TADF complex DPTZ-DBO2 fluorescence emission intensity count and PD-10 fraction number measured using spectrofluorometer, excitation 488 nm, emission 495-595 nm, taking intensity count at 515 nm. From liposome formation protocol with 5 mM TADF fluorophore. Each marker represents an individual emission spectrum from which the intensity count at 515 nm was obtained. A smooth line links the markers.

**Supplementary Figure 3.** Comparison of (**a-b**) liposome packaged and (**c**) free CF, showing liposome packaged CF, from fraction 1, imaged (**a**) 30 minutes and (**b**) 7 days after PD-10 column separation. Packaged and free CF gave the same fluorescence spectroscopy fluorescence intensity count. Scale bar (bottom right, white rectangle) 25 μm in length.

**Supplementary Figure 4.** The effect of freeze-pump-thaw on the integrity of liposomal CF, comparing the physical appearance of CF-containing liposomes (**left panel**) before and (**right panel**) after freeze-pump-thaw treatment, after PD-10 column separation. Liposomes formed with 5 mM CF, 100 nm in diameter. PD-10 fraction 1 imaged. Scale bar (bottom right, white rectangle) 25 μm in length.

**Supplementary Figure 5.** NIH 3T3 cellular introduction of (**upper row**) non-functionalised and (**lower row**) amine-functionalised TADF complex DPTZ-DBO2 doped polystyrene nanoparticles with 24 hr incubation time at 100 μg mL^-1^ final concentration. (**upper row**) Non-functionalised nanoparticles ~40 nm in diameter, (**lower row**) amide-functionalised nanoparticles ~100 nm in diameter. Confocal microscopy images for (**A**) Hoechst 33342 emission of nuclei, (**B**) DPTZ-DBO2 emission, (**C**) Hoechst 33342 and DPTZ-DBO2 emission overlaid, and (**D**) bright field. Scale bar (bottom right, white rectangle) 25 μm in length.

**Supplementary Figure 6.** HepG2 cellular introduction of liposomal CF trialling incubation times of (**uppermost row**) 4 hrs, (**2^nd^ row from top**) 8 hrs, (**3^rd^ row from top**) 16 hrs and (**bottom row**) 24 hrs with 20% v/v PD-10 column separated liposomal CF, with liposomes 100 nm in diameter (see figure SI 7 for cellular introduction trialling 40% v/v liposome solution concentration and figure SI 8 for 400 nm liposome diameter). Confocal microscopy images for (**A**) Hoechst 33342 emission of nuclei, (**B**) CF emission, (**C**) Hoechst 33342 and CF emission overlaid, and (**D**) bright field. Scale bar (bottom right, white rectangle) 25 μm in length.

**Supplementary Figure 7.** HepG2 cellular introduction of liposomal CF trialling liposomal CF solution concentrations of (**top row**) 20% v/v cell volume, (**bottom row**) 40% v/v cell volume, incubated for 24 hrs with 100 nm diameter liposomes. Confocal microscopy images for (**A**) Hoechst 33342 emission of nuclei, (**B**) CF emission, (**C**) Hoechst 33342 and CF emission overlaid, and (**D**) bright field. Scale bar (bottom right, white rectangle) 25 μm in length.

**Supplementary Figure 8.** HepG2 cellular introduction of liposomal CF trialling liposome diameters of (**top two rows**) 100 nm, (**bottom two rows**) 400 nm, incubated for 24 hrs with (**uppermost row and third from top**) 20% v/v or (**second row from top and bottom row**) 40% v/v PD-10 desalting column separated liposomes. Confocal microscopy images for (**A**) Hoechst 33342 emission of nuclei, (**B**) CF emission, (**C**) Hoechst 33342 and CF emission overlaid, and (**D**) bright field. Scale bar (bottom right, white rectangle) 25 μm.

**Supplementary Figure 9.** HepG2 cellular introduction repeats of liposomal DPTZ-DBO2 under 24 hr incubation with 20% v/v PD-10 desalting column separated liposomes from fraction 1, with liposomes 100 nm in diameter. Confocal microscopy images for (**A**) DPTZ-DBTO2 emission, (**B**) bright-field and (**C**) DPTZ-DBTO2 emission and bright-field overlaid. Scale bar (bottom right, white rectangle) 25 μm in length.

**Supplementary Figure 10.** Time-resolved fluorescence microscopy showing an extended time window. At time = 0 ns, the excitation pulse excite the sample material, leading to decay of the fluorescence fitted as described earlier. At negative times, the tail end of the decaying fluorescence from the previous excitation event is shown. A dashed line indicates a baseline drawn from an average count of the signal around time *t* = 0 ns. The pulse repetition rate of the laser systems was 1 MHz which gives a 1 μs delay time between pulses. The upper panel shows liposomal DPTZ-DBO2 in HepG2 cells, and the lower panel shows DPTZ-DBO2 nanoparticles in NIH 3T3 cells.

It is noteworthy that the intensity at < – 60 ns is clearly higher for the liposomal DPTZ-DBO2 (upper panel) as compared to DPTZ-DBO2 nanoparticles. The emission detected at < – 60 ns is occurring > 900 ns after excitation.
